# The Mediterranean Shores of America

**Published:** 1892-05

**Authors:** 


THE MEDITERRANEAN SHORES OF AMERICA. Southern California, its climatic, physical, and meteorological conditions. By P. C. Remondino, M. D. (Jefferson), Member of the American Medical Association, of the American Public Health Association, of the San Diego County Medical Society, of the State Board of Health of California; Vice-President of the California State Medical Society, and of the Southern California Medical Society, etc., etc. Fully illustrated. Philadelphia and London: The F. A. Davis Co., Publishers, 1892.

This book, one octavo volume, one hundred and sixty pages, forty-four illustrations and map, treats of the wonderful climate of Southern California, its peculiar temperature and atmospheric humidity; of its climatology as fully varied as that of the north of Italy, but on the contrary these extremes being favorable to health and long life. It is intended as a short guide or handbook to the seeker after climate for health. It is almost all devoted to the effects of the California climate on those suffering from pulmonary diseases. It is full of valuable statistics relating to those diseases, being a most striking evidence of the author's deep and varied study of the subject. Of the influence of the climate he says: "Those who experience the greatest immediate benefit are those whom some serious illness has left weakly and broken down, the wrecks of over-work and malaria; the nervous and anæmic, and those afflicted with some mild disease of the respiratory organs." Of phthisis, he continues, in another part: "The climate can arrest diseased action, in certain cases prevent its development, in others it can even prolong the days of the organically demoralized, but it cannot reanimate the mummified remains of Rameses II any more than it can reconstruct new organs where they have undergone a complete structural change or suppurative destruction. There is a time when an invalid can come with what might be said every chance for a recovery in his favor; but he must come before the undertows of malassimilation, malnutrition, and general destruction have carried him off his feet or before all recuperative powers are completely exhausted." He also cautions the invalid seeking for health from the fatigue and over-work of the tourist. The illustrations are of the principal hotels; the old missions of the Spanish priests; of the vegetation of the country, of natural scenes, and of the aboriginal natives. The work, on the whole, is valuable and instructive, and well worth recommendation to those interested in the subject. H. P.

